# Does imaging correctly influence the surgical management in the setting of neoadjuvant chemotherapy?

**DOI:** 10.1186/bcr3286

**Published:** 2012-11-09

**Authors:** H Fatayer, B Kim, N Sharma, DD Manuel, BJG Dall

**Affiliations:** 1 Leeds Teaching Hospitals NHS Trust, Leeds, UK

## Objective

To evaluate the role of imaging in aiding surgical management and assess whether imaging resulted in unnecessary mastectomies.

## Methods

A retrospective analysis of 165 patients with invasive breast cancer treated with neoadjuvant chemotherapy (NAC) (2005 to 2009). Bilateral cancers were analysed separately (total = 169). Data collected include tumour extent on mammogram and MRI, radiology advice for surgery, surgery performed, and whole tumour size on postsurgical specimens. MRI report recommended wide-local excision (WLE), mastectomy, or WLE with multidisciplinary team discussion (MDTD) based on imaging. We used residual tumour size <40 mm on pathology as a cut-off for suitability of WLE.

## Results

Radiology advice regarding surgery was suggested in 167 cases and advice was followed in 142 (85%) cases. Fifty-nine cases were suggested for WLE; 46 (78%) underwent WLE with six requiring further surgery (no further disease in four) and 13 (22%) underwent mastectomy based on patient's choice or clinical decision. Ninety-one cases were suggested for mastectomy; two (2%) had successful WLE due to patient's choice (one had therapeutic mammoplasty) and 89 (98%) had mastectomy. Seventy-four were appropriate (inflammatory, multicentric, pathology ≥40 mm, patient choice or surgical cosmesis) and based on pathology 15 may have had inappropriate mastectomies as residual disease <40 mm. Seventeen cases were considered for WLE with MDTD, seven (41%) had successful WLE and 10 (59%) had mastectomy; all were appropriately advised when compared with final pathology.

## Conclusion

This series suggests that NAC offers more opportunities for breast conservation than are being realised. Multifocal disease and extensive calcifications may not mandate mastectomy if the patient responds well to chemotherapy.

